# Engineering *Yarrowia lipolytica* for the sustainable production of β-farnesene from waste oil feedstock

**DOI:** 10.1186/s13068-022-02201-2

**Published:** 2022-10-03

**Authors:** Yinghang Liu, Jin Zhang, Qingbin Li, Zhaoxuan Wang, Zhiyong Cui, Tianyuan Su, Xuemei Lu, Qingsheng Qi, Jin Hou

**Affiliations:** State Key Laboratory of Microbial Technology, Shandong University, Binhai Road 72, Qingdao, 266237 China

**Keywords:** β-Farnesene, β-Oxidation, Lipid metabolism, Waste lipid feedstock, *Yarrowia lipolytica*

## Abstract

**Background:**

β-Farnesene is a sesquiterpene with versatile industrial applications. The production of β-farnesene from waste lipid feedstock is an attractive method for sustainable production and recycling waste oil. *Yarrowia lipolytica* is an unconventional oleaginous yeast, which can use lipid feedstock and has great potential to synthesize acetyl-CoA-derived chemicals.

**Results:**

In this study, we engineered *Y. lipolytica* to produce β-farnesene from lipid feedstock. To direct the flux of acetyl-CoA, which is generated from lipid β-oxidation, to β-farnesene synthesis, the mevalonate synthesis pathway was compartmentalized into peroxisomes. β-Farnesene production was then engineered by the protein engineering of β-farnesene synthase and pathway engineering. The regulation of lipid metabolism by enhancing β-oxidation and eliminating intracellular lipid synthesis was further performed to improve the β-farnesene synthesis. As a result, the final β-farnesene production with bio-engineering reached 35.2 g/L and 31.9 g/L using oleic acid and waste cooking oil, respectively, which are the highest β-farnesene titers reported in *Y. lipolytica*.

**Conclusions:**

This study demonstrates that engineered *Y. lipolytica* could realize the sustainable production of value-added acetyl-CoA-derived chemicals from waste lipid feedstock.

**Supplementary Information:**

The online version contains supplementary material available at 10.1186/s13068-022-02201-2.

## Introduction

Waste cooking oil (WCO) is a waste product from deep-frying oil, containing triglycerides, polar compounds, and nonvolatile compounds. Currently, the annual global consumption of vegetable oil is approximately 200 million tons, with 32% of the edible oil used becoming waste [1]. Most WCO is dumped into sewage networks, causing the pollution of water bodies [2]. Therefore, the recycling and reutilization of waste oil as a feedstock for the production of value-added chemicals are desirable to prevent environmental pollution, and will also increase the economic value of the WCO. WCO has been used to produce biodiesel, bioplastics, citric acid, and erythritol, providing potential economical and eco-friendly ways to recycle WCO [3–5].

*Yarrowia lipolytica* was first isolated from a lipid-rich environment because of its excellent lipid degradation ability. *Y. lipolytica* contains 16 lipase coding genes that enable this yeast to decompose lipids intracellularly and extracellularly [6]. Another prominent characteristic of *Y. lipolytica* is its strong β-oxidation ability, which enables the decomposition of fatty acids into acetyl-CoA. Therefore, *Y. lipolytica* has the potential capability to synthesize acetyl-CoA-derived chemicals, such as terpenoids and polyketides, using lipid feedstock.

Farnesene is an acyclic volatile sesquiterpene constituent of several plant essential oils that has two isomers: α-farnesene and β-farnesene [7]. Due to the properties of β-farnesene, it has been widely applied in the industrial and agricultural production of pesticides, lubricants, surfactants, cosmetics, and biofuels [8]. Recently, β-farnesene has been used to synthesize vitamin E [9]. Microbial synthesis is an ideal alternative for farnesene production because it is cost-effective, eco-friendly, and sustainable in terms of its production, compared with natural extraction and chemical synthesis. Several studies have engineered microorganisms for farnesene production [8]. The highest β-farnesene titer was approximately 10.31 g/L in *Escherichia coli* [10]. Using glucose as feedstock, the β-farnesene production reached 130 g/L in *Saccharomyces cerevisiae* [11]. Our recent work indicated that *Y. lipolytica* is also a potential farnesene production strain, and α-farnesene could be produced at 25.5 g/L [12]. However, all the strategies described above used sugar as the feedstock [11, 12], and there are few studies on the use of lipid feedstock to produce farnesene [13].

Farnesene is composed of isoprene units biochemically synthesized in eukaryotes from three acetyl-CoA molecules via the mevalonate pathway. When glucose is used, the synthesis of acetyl-CoA generates CO_2_, which leads to carbon loss, whereas the use of fatty acids as a feedstock results in no carbon loss in acetyl-CoA synthesis. The farnesene theoretical conversion rate for oleic acid is 0.723 g/g, which is higher than that for glucose (0.252 g/g). Hence, compared with using glucose feedstocks, it is possible to obtain higher yields by producing farnesene from lipid feedstocks using *Y. lipolytica*.

**Fig. 1:**
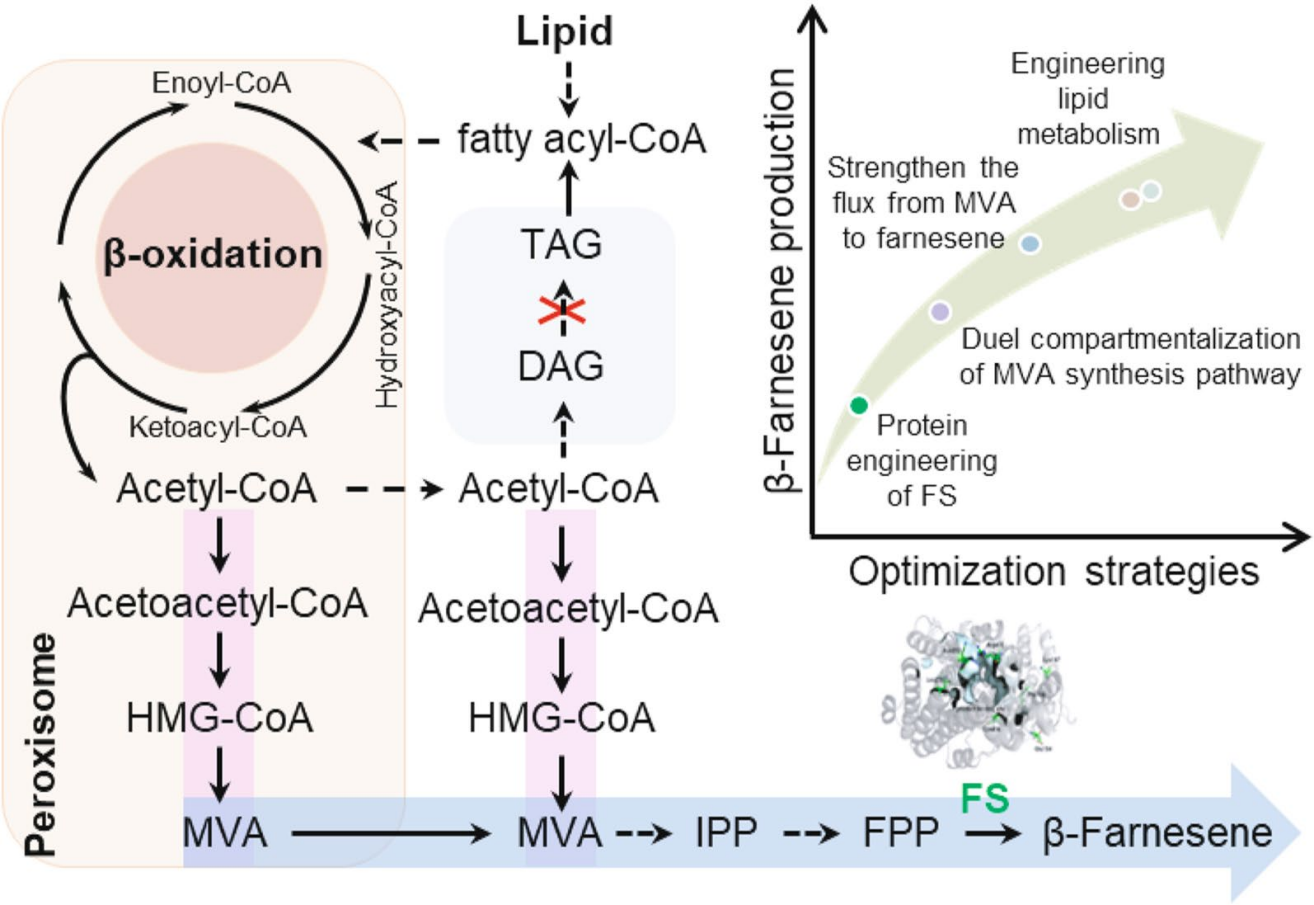
Engineering *Yarrowia lipolytica* for β-farnesene production from lipids. The mevalonate synthetic pathway was dual compartmentalized in both peroxisomes and the cytoplasm. The catalytic efficiency of β-farnesene synthase was improved by protein engineering. The lipid metabolism and β-farnesene synthetic pathway were regulated to improve β-farnesene production

In this study, we engineered *Y. lipolytica* for the efficient production of high value-added β-farnesene from WCO. The lipid metabolism, mevalonate pathway, and farnesene synthase were systematically engineered to improve β-farnesene production (Fig. 1). After fermentation optimization, the highest reported titer of β-farnesene was realized using oleic acid or WCO as feedstock in *Y. lipolytica*. Our work demonstrated that by regulating fatty acid metabolism, the engineered *Y. lipolytica* could efficiently synthesize β-farnesene or other acetyl-CoA-derived chemicals from WCO. This study will contribute to the recycling of WCO for value-added chemical production.

## Results and discussion

### Peroxisome compartmentalization directed the acetyl-CoA flux to the mevalonate pathway

**Fig. 2:**
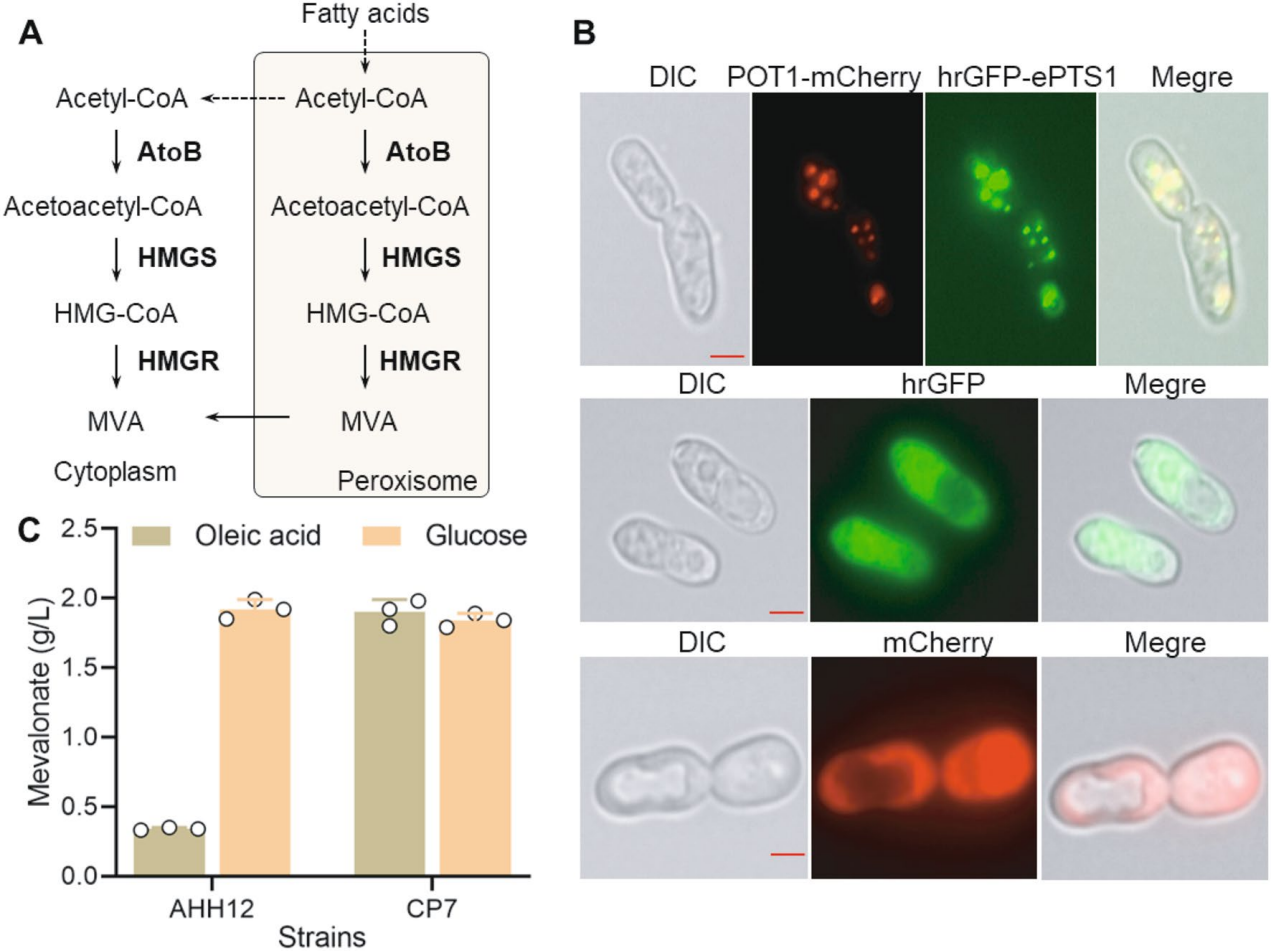
Peroxisome and cytoplasm dual compartmentalization of mevalonate synthetic pathway to improve mevalonate production from lipids. **A** The mevalonate synthetic pathway was dual compartmentalized in both peroxisomes and the cytoplasm. **B** The localization of the ePTS1 signal peptide in *Y. lipolytica*. The ePTS1 signal peptide was fused to the *C*-terminus of hrGFP. The peroxisome protein POT1 was fused with mCherry as a peroxisome localization marker. **C** The mevalonate production of different strains using oleic acid and glucose. Data represent the mean ± SD of three biological replicates

Previously, we constructed a mevalonate producer, AHH12, by overexpressing *AtoB*, *HMGR*, and *HMGS*, which could produce 1.96 g/L mevalonate using glucose as the carbon source [12]. However, when using oleic acid as the carbon source, AHH12 only produced 0.34 g/L mevalonate (Fig. 2C), much lower than the yield with glucose. These results demonstrated that the overexpression of the mevalonate pathway in the cytosol could not effectively direct the flux to mevalonate synthesis when using lipid feedstock. The β-oxidation of fatty acids occurs in the peroxisome and generates acetyl-CoA as the end product (Fig. 2A). Acetyl-CoA cannot be directly transported from peroxisomes to the cytosol, which generally requires a glyoxylate shunt to synthesize carboxylic acids or alternatively is transported to the carnitine/acetyl-carnitine shuttle for further use [14]. To directly use peroxisome acetyl-CoA for terpenoid production, the enzymes involved in mevalonate synthesis were directly targeted to peroxisomes (Fig. 2A). An enhanced targeting tag (ePTS1) has been previously developed in *S. cerevisiae* [15], and its localization efficiency was verified in *Y. lipolytica*. Therefore, we fused the ePTS1 signal at the *C*-terminus of hrGFP and observed the protein localization. The peroxisome protein POT1 fused with mCherry was used as a peroxisome localization marker [16]. The results of fluorescence microscopy indicated that hrGFP fused with the ePTS1 signal peptide was localized in the peroxisomes with high efficiency (Fig. 2B). Subsequently, we fused the ePTS1 signal peptide with the *C*-termini of AtoB, HMGR, and HMGS and expressed these proteins in AHH12 for dual-compartment localization in both peroxisomes and the cytoplasm. The results indicated that this strain (CP7) accumulated 1.90 g/L mevalonate after 96 h of fermentation using oleic acid as the carbon source, which was 5.3-fold higher than the AHH12 strain. Notably, CP7 accumulated 1.84 g/L mevalonate, which is consistent with the 1.92 g/L observed for the AHH12 strain when using glucose as the carbon source (Fig. 2C), indicating that the CP7 strain can be used as a platform for terpenoid production, including β-farnesene. These results demonstrated that targeting the mevalonate synthesis pathway in peroxisomes is an efficient strategy to improve the efficiency of converting peroxisome acetyl-CoA to mevalonate when using lipid as the feedstock in *Y. lipolytica*. Consistent with previous studies, our work showed that subcellular metabolic engineering offers an effective way for terpenoid synthesis [17, 18]. Mevalonate is a small molecule that can freely diffuse from the peroxisome to the cytoplasm. To avoid an excessive increase in the burden of peroxisome, we did not locate the downstream mevalonate pathway in the peroxisome.

### Engineering the activity of β-farnesene synthases

The activity of the β-farnesene synthase plays a key role in microbial heterologous synthesis of β-farnesene. We expressed β-farnesene synthases from different sources in *Y. lipolytica*. A total of 11 β-farnesene synthases were first selected to construct the evolutionary tree (Additional file 1: Figure S1A). β-Farnesene synthases distributed in different evolutionary tree branches, including *Mentha asiatica*, *Handroanthus impetiginosus*, *Artemisia annua*, *Pseudotsuga menziesii,* and *Striga asiatica*, were further selected, codon-optimized, and expressed in *Y. lipolytica*. Interestingly, only β-farnesene synthase from *A. annua* (AanFS) produced detectable β-farnesene using glucose as the carbon source (Additional file 1: Figure S1B). Farnesene synthase from soybean or *M. domestica* has been reported to show better catalytic activity in *S. cerevisiae* or *P. pastoris*, respectively [19, 20]. Therefore, β-farnesene synthases appeared to exhibit different activities in different hosts, and the issues of protein activity and protein expression may both affect its heterologous expression, therefore screening terpenoid synthases from different sources can obtain high-activity synthases.

**Fig. 3:**
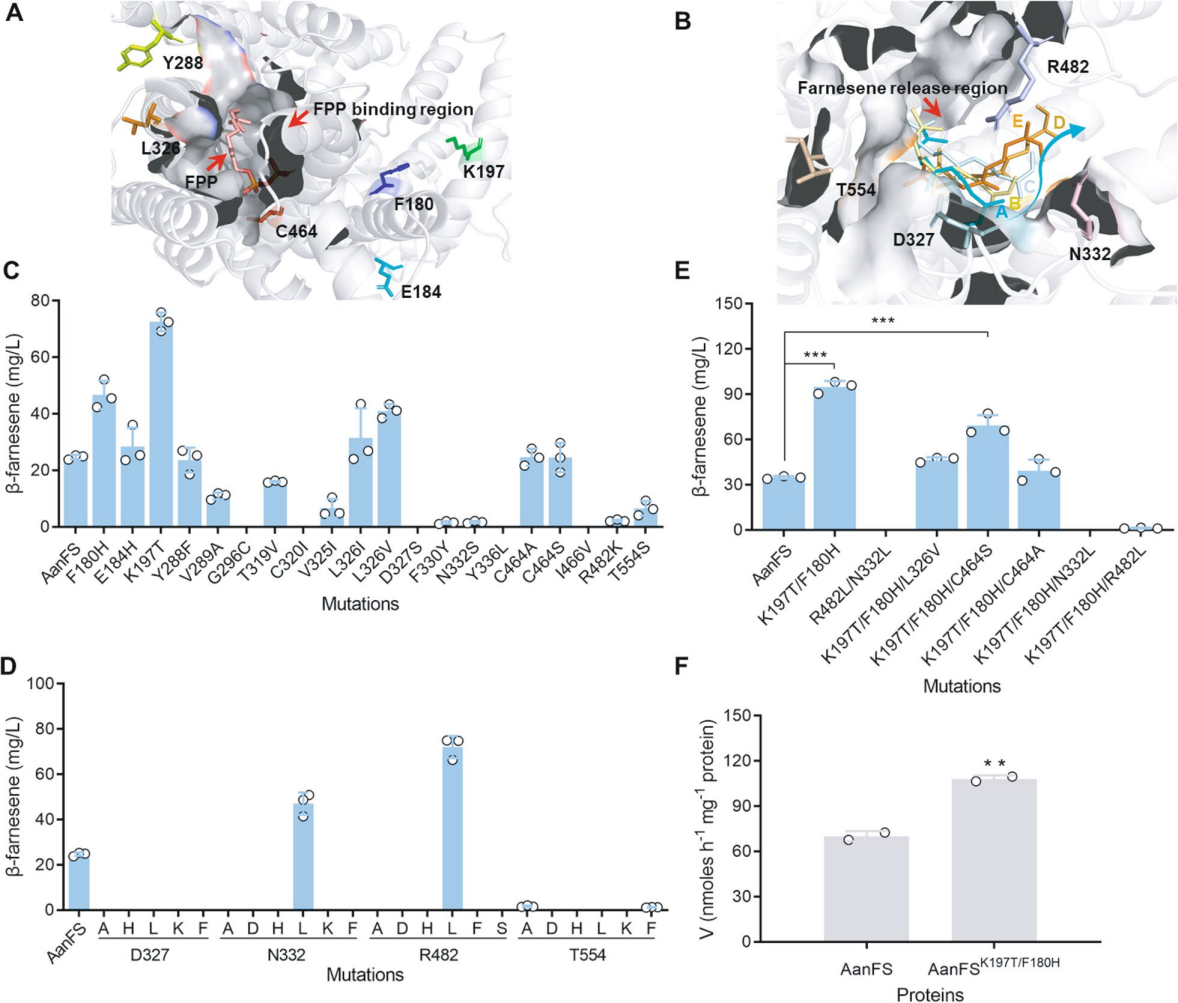
Protein engineering to improve the β-farnesene synthase activity. **A** The amino acids in the catalytic active center were engineered to improve the catalytic activity. **B** The amino acids in the β-farnesene-release region were designed to enhance β-farnesene release. β-Farnesene moves from A to E in the product-release region. **C** β-Farnesene production after amino acid mutations in the catalytic active center. **D** β-Farnesene production after amino acid mutations in the β-farnesene-release region. **E** β-Farnesene production after a combination of the beneficial mutations. **F** In vitro activity of the mutant AanFS^K197T/F180H^. Data represent the mean ± SD of two or three biological replicates. Statistical analysis was performed by using one-way ANOVA (** *p* < 0.05, *** *p* < 0.01)

To improve β-farnesene production, we employed structure-guided protein engineering to enhance the catalytic efficiency of AanFS. Homologous modeling was used to create a model of the three-dimensional structure of β-farnesene synthase. We constructed a three-dimensional structure model of AanFS to construct the AanFS-FPP complex and identified key amino acids around the FPP binding region (F180, E184, K197, Y288, V289, G296, T319, C320, V325, L326, D327, F330, N332, Y336, C464, I466, R482, and T554) (Fig. 3A). Using HotSpot Wizard 3.0, the mutations of the above amino acid (F180H, E184H, K197T, Y288F, V289A, G296C, T319V, C320I V325I, L326I, L326V, D327S, F330Y, N332S, Y336L, C464A, C464S, I466V, R482K, and T554S) were given and then they were introduced into AanFS and expressed in *Y. lipolytica*. The results showed that mutations of F180H, E184H, K197T, L326I, and L326V improved the β-farnesene titers by 89.1%, 15.0%, 193.9%, 27.5%, and 66.0%, respectively (Fig. 3C).

In addition to the FPP binding region, we determined the effect of the farnesene-release region on the catalytic efficiency of farnesene synthase [21]. Therefore, the amino acid residues in the product-release region of farnesene synthase were also engineered as the second target. According to the analysis of the MD simulations of the trajectory of the AanFS-farnesene structure, D327, N332, R482, and T554 were determined to be the key amino acid residues that affect the release of farnesene (Fig. 3B). These four amino acid residues were then replaced by Phe, His, Lys, Asp, Leu, Ser, and Ala, which contain different side-chain types. The β-farnesene production was compared with AanFS and only N332L and R482L mutations improved the β-farnesene production, by 34% and 102.2%, respectively (Fig. 3D). These two substitutions may prevent the formation of ionic bonds in the release region of β-farnesene, which might accelerate the release of this compound. In contrast, the other mutations almost abolished β-farnesene synthesis.

To further improve the catalytic efficiency of β-farnesene synthase, the beneficial mutations were combined. The K197T/F180H mutation had the highest activity and the β-farnesene titer improved by 283.8% compared with AanFS (Fig. 3E). This titer was also 30.7% higher than that of the K197T single mutation. We further purified the protein AanFS^K197T/F180H^ (Additional file 1: Figure S2) and determined the catalytic activity for the synthesis of β-farnesene from FPP in vitro. Consistent with the in vivo activity, the catalytic efficiency of AanFS^K197T/F180H^ reached 108.1 nmol/h/mg protein in vitro, which was 54.4% higher than that in the protein of AanFS (70.1 nmol/h/mg) (Fig. 3F). These data indicated that the catalytic efficiency of AanFS could be improved by introducing the K197T/F180H mutation. Although 54.4% higher activity was observed, the β-farnesene titer improved by 283.8% compared with AanFS in *Y. lipolytica*. As the folding of heterologous proteins often affect their expression efficiency, the identified mutation may increase both activity and expression of the enzyme. Interestingly, the lack of β-farnesene production in the strains containing mutations in the farnesene-release region was attributed to the fragility of AanFS caused by the N332L and R482L mutations.

### Strengthening the flux from mevalonate to β-farnesene

**Fig. 4:**
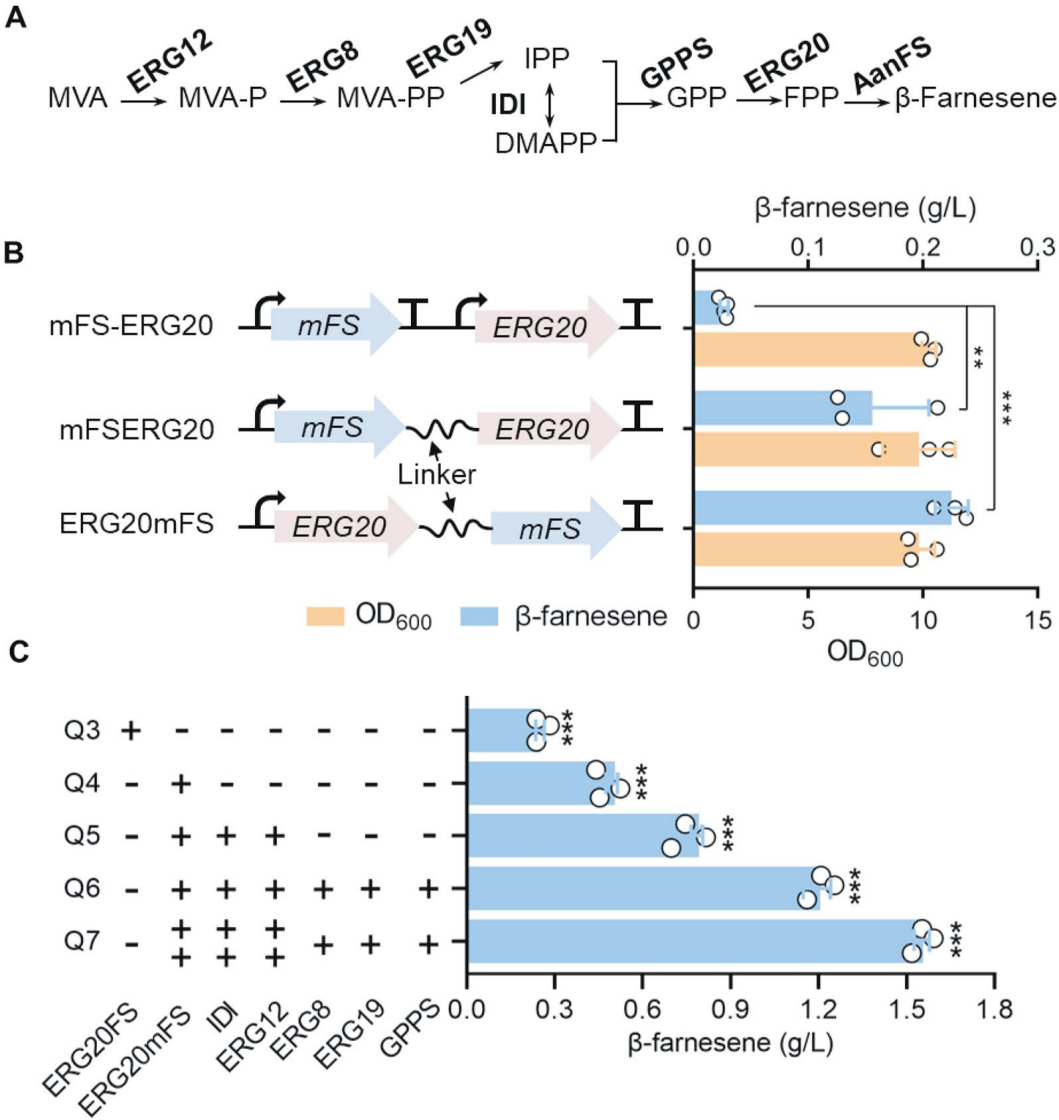
Engineering the downstream mevalonate pathway to improve the β-farnesene production. **A** Metabolic pathway from mevalonate to β-farnesene. **B** The β-farnesene production of the strains containing different fusion forms of *ERG20* and *AanFS*^*K197T/F180H*^. **C** Overexpression of the genes encoding the enzymes in the mevalonate to β-farnesene pathway improved the β-farnesene production. *mFS* represents *AanFS*^*K197T/F180H*^. Data represent the mean ± SD of three biological replicates. Statistical analysis was performed by using one-way ANOVA (**p < 0.05, ***p < 0.01)

Enzymes in the synthesis pathway from mevalonate to β-farnesene were overexpressed to optimize the β-farnesene synthesis in *Y. lipolytica* (Fig. 4A). The push–pull restriction strategy is an effective method for improving the yield of the required products. In the present study, AanFS^K197T/F180H^ and ERG20 were fused to enhance the downstream metabolic intensity and direct the metabolic flow toward β-farnesene synthesis. To avoid adverse effects of the fusion on the activities of AanFS^K197T/F180H^ and ERG20, we added a GGGGS linker between the moieties and compared the β-farnesene production of constructs with different fusion orders through episomal plasmid expression [22, 23] (Fig. 4B). The results showed that the β-farnesene titer was improved 3.3- and 7.3-fold with the fusion constructs AanFS^K197T/F180H^ERG20 and ERG20AanFS^K197T/F180H^, respectively, when compared with the individual expression of ERG20 and AanFS^K197T/F180H^.

*ERG20AanFS*^*K197T/F180H*^ and *ERG20AanFS* were integrated into the mevalonate overproduction strain CP7. A titer of 0.52 g/L β-farnesene was obtained with the *ERG20AanFS*^*K197T/F180H*^ expression strain (Q4), which was 2.1-fold higher than the titer of 0.25 g/L with the *ERG20AanFS* expression strain (Q3) (Fig. 4C). We overexpressed the rate-limiting enzymes, IDI1 and ERG12, of the mevalonate pathway in strain Q4 to obtain strain Q5. The β-farnesene production of the Q5 strain reached 0.79 g/L. Then, the rest of the enzymes in the mevalonate pathway including ERG8, ERG19 and GPPS were overexpressed. As a result, the β-farnesene production was further increased to 1.21 g/L (Q6 strain). Increasing the copy number of key enzymes in the mevalonate pathway can further improve the production of terpenoids [24]. Therefore, *ERG12*, *IDI* and *ERG20AanFS*^*K197T/F180H*^ were reintegrated into the genome of the Q6 strain to obtain the Q7 strain. The β-farnesene production was further increased by 28.9% and reached 1.56 g/L (Fig. 4C). In addition, we determined the relative transcriptional levels of *AanFS*^*K197T/F180H*^ in the Q6 and Q7 strains, and the result showed that the expression level of *AanFS*^*K197T/F180H*^ in Q7 was increased by 4.6-fold compared with Q6. The copy number of *AanFS*^*K197T/F180H*^ in Q7 was 9, which was higher than the 6 in the Q6 strain (Additional file 1: Figure S3).

### Engineering the β-oxidation in lipid metabolism

**Fig. 5:**
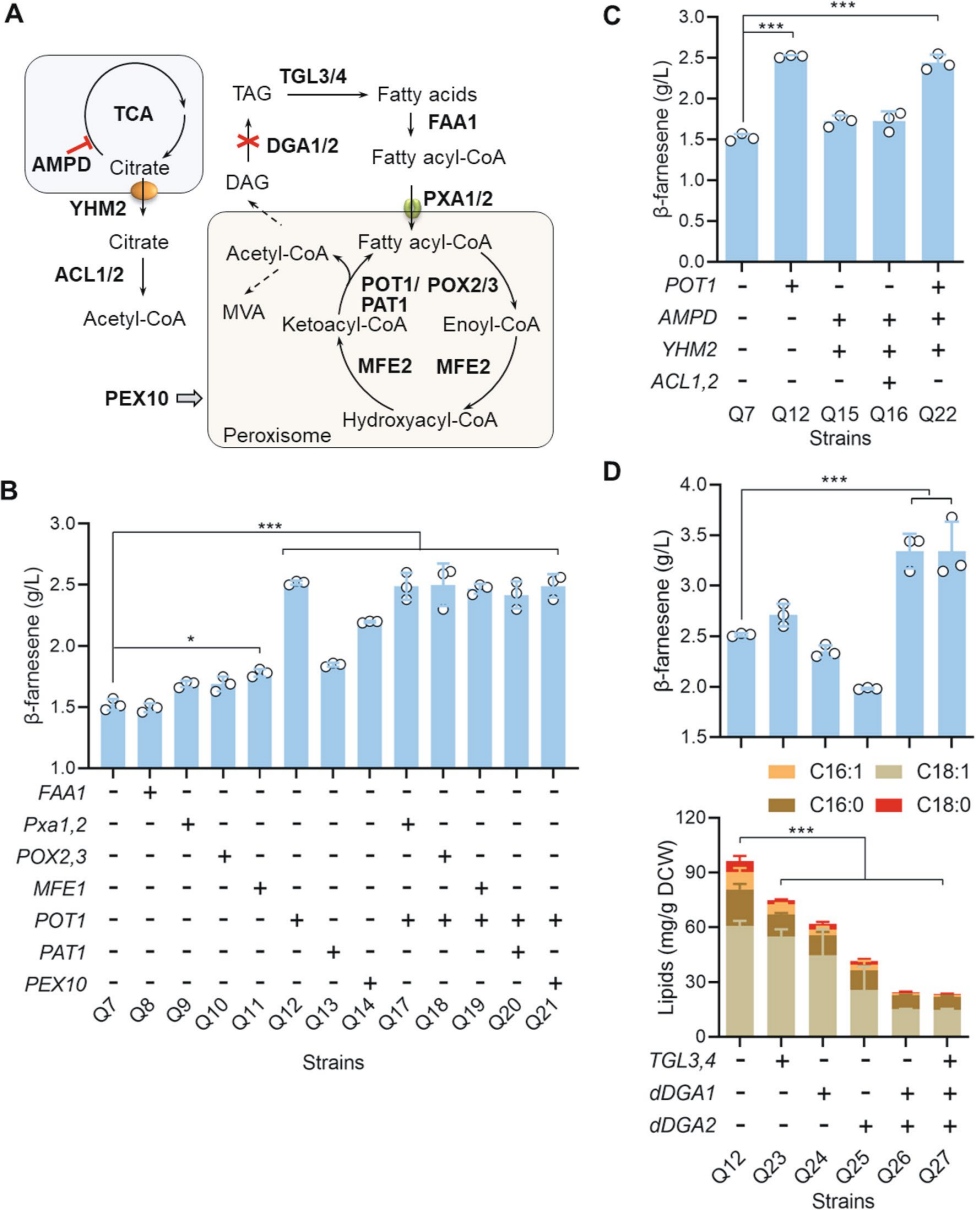
Engineering β-oxidation and lipid metabolism to improve the β-farnesene production. **A** β-Oxidation and lipid metabolic pathways. **B** Effect of overexpression of the genes involved in β-oxidation on β-farnesene production. **C** Effect of engineering the cytosolic acetyl-CoA metabolism on β-farnesene production. **D** Regulating the lipid metabolism improved β-farnesene production and reduced intracellular lipid accumulation. Data represent the mean ± SD of three biological replicates. Statistical analysis was performed by using one-way ANOVA (*p < 0.1, ***p < 0.01)

*Y. lipolytica* converts fatty acids to acetyl-CoA through β-oxidation, which requires the participation of a series of enzymes (Fig. 5A). In order to provide more acetyl-CoA for β-farnesene synthesis, we overexpressed the genes related to the fatty acid utilization in Q7 strain (Additional file 1: Figure S6). Previous study showed *S. cerevisiae* possesses four fatty acid acyl-CoA synthases that activate fatty acids with different lengths before entering peroxisomes, there was only one acyl-CoA synthase (FAA1) found in the cytoplasm of *Y. lipolytica* [25]. Then *FAA1* was overexpressed in Q7 strain. However, overexpression of *FAA1* (Q8) did not improve the β-farnesene production (Fig. 5B), indicating that the reaction catalyzed by FAA1 might not be the limiting step for fatty acid use. A previous study showed that peroxisomal acyl-CoA transporters PXA1 and PXA2 are responsible for transporting the activated fatty acyl-CoA to peroxisomes in *Y. lipolytica* [25]. Simultaneous *PXA1* and *PXA2* overexpression (Q9) enhanced β-farnesene production by 12.8%, reaching a titer of 1.68 g/L. Therefore, enhancement of the transport of fatty acyl-CoA to peroxisomes can also reduce its storage in lipid droplets [25]. We next overexpressed the enzymes involved in β-oxidation, including the long-chain fatty acyl-CoA oxidases POX2 and POX3, the peroxisomal multifunctional enzyme MFE2, and the 3-ketoacyl-CoA thiolase POT1 in Q7 strain, and the result showed that β-farnesene production from the generated strains was improved by 13% (Q10), 20% (Q11) and 69% (Q12), respectively. Notably, we found that the *POT1* overexpression strain (Q12) exhibited the highest β-farnesene titer to 2.52 g/L among these overexpression strains (Fig. 5B). As reported by Ma et al. overexpression of *POT1* significantly improved α-bisabolol production compared with the overexpression of *POX1-6* or *MFE2* [26].

We also explored the effects of PAT1 overexpression, which catalyzes the reversible thiolytic cleavage of 3-ketoacyl-CoA into acyl-CoA and acetyl-CoA in Q7 [27]. As a result, the β-farnesene titer was increased by 24.2% (Q13) (Fig. 5B). The peroxisome biogenesis factor 10 (PEX10) is often used as a regulator of peroxisomes and has been shown to improve the production of many acetyl-CoA-derived chemicals [28]. Here, we found that overexpression of *PEX10* in Q7 strain promoted β-farnesene synthesis as well, and increased the β-farnesene titer by 48.3% (Q14) (Fig. 5B). The accumulation of β-farnesene may be caused by the enhanced degradation and use of triacylglycerides (TAGs) [29, 30] or impacting the number and morphology of the peroxisomes [31]. As *POT1* overexpression strain (Q12) showed highest β-farnesene production capability, the *POX2*, *POX3*, *MFE1*, *PAT1*, and *PEX10* genes were over-expressed in Q12 strain to investigate the combination effect. However, compared with Q12 strain, β-farnesene production of these strains did not improve significantly (Fig. 5B).

In addition to enhancing the lipid usage ability, we also engineered the citric acid metabolism and lipid anabolism to regulate the flow of acetyl-CoA toward the synthesis of β-farnesene. To direct more cytosolic acetyl-CoA flow toward β-farnesene synthesis, we further overexpressed AMP deaminase (AMPD), which represses the activity of isocitrate dehydrogenase in the TCA cycle, and the mitochondrial citrate carrier YHM2, which transports citric acid from the mitochondria to the cytoplasm, in Q7 strain [32, 33]. The generated recombinant strain Q15 was found to produce a titer of 1.72 g/L β-farnesene, with an improvement of 15.4% compared to the Q7 strain (Fig. 5C). However, further overexpression of the ATP-dependent citrate lyases ACL1 and ACL2 in the Q15 strain, which could enhance the conversion of citric acid in the cytoplasm to acetyl-CoA, did not further increase the β-farnesene production of the recombinant Q16 strain. These results demonstrated that the activities of endogenous ACL1 and ACL2 were sufficient for cytosolic acetyl-CoA synthesis. We also overexpressed *AMPD* and *YHM2* in the *POT1* overexpression strain Q12, but no further improvement in the β-farnesene production was observed (Q22) (Fig. 5C). These results indicated that POT1 is the most critical limiting factor in the fatty acid usage module. We therefore used *POT1* overexpressing strain Q12 for further engineering.

### Diverting acetyl-CoA flux from lipid synthesis toward β-farnesene production

Previous study has shown that *Y. lipolytica* has strong lipid anabolism, which consumes a considerable amount of the carbon source and reduces the flux toward product synthesis [34]. Therefore, we determined the accumulation of lipids in different *Y. lipolytica* strains. As shown in Fig. 5D, Additional file 1: Figure S4, when oleic acid was used as the carbon source, the lipid accumulation of the Q12 strain was 96.3 mg/g DCW, which was lower than that of the CP7 strain (142.9 mg/g DCW). This result indicated that the enhanced metabolism of the β-farnesene synthesis pathway of the Q12 strain reduced the metabolic flux toward lipid synthesis. Similarly, because of the enhancement of mevalonate synthesis, the accumulation of major lipids in the CP7 strain was lower than the 179.8 mg/g DCW observed in the PO1f strain (Additional file 1: Figure S4).

We then engineered the biosynthesis to direct the flow from intracellular lipid accumulation toward the β-farnesene synthesis pathway. The lipids in *Y. lipolytica* are mainly stored in lipid droplets as TAGs [35]. Triacylglycerol (TAG) lipase TGL4, which requires the activation of TGL3, can release fatty acids from TAGs [36]. The inactivation of *TGL3/4* usually leads to the accumulation of lipids in *Y. lipolytica* [37]. Here, in this study we overexpressed *TGL3* and *TGL4* in the Q12 strain, and found that the lipid content in this generated strain (Q23) decreased to 74.8 mg/g DCW, while the titer of β-farnesene increased to 2.71 g/L, which was 8% higher than that of the Q12 strain (Fig. 5D).

In addition to promoting fatty acid release, we also generated the strains with reduced TAG synthesis. The intracellular lipid accumulation has been shown to be decreased when the diacylglycerol acyltransferases *DGA1* or *DGA2*, which catalyze the formation of TAGs from diacylglycerol (DAGs), was knocked out [38]. Here, we found that when *DGA1* and *DGA2* were separately deleted in the Q12 strain using the CRISPR–Cas9 system, the lipid accumulation decreased to 62.1 (Q24) and 41.5 mg/g DCW (Q25), respectively (Fig. 5D). However, the single gene deletion of *DGA1* or *DGA2* did not increase the β-farnesene production. Interestingly, the simultaneous deletion of *DGA1* and *DGA2* (Q26 strain) reduced the lipid accumulation to 24.1 mg/g DCW, and the β-farnesene titer reached 3.34 g/L, which was 33.1% higher than that of Q12. In addition, we overexpressed *TGL3* and *TGL4* in Q26 strain to increase β-farnesene production. The recombinant Q27 strain had similar β-farnesene production compared to the Q26 strain, which might be attributed to the relatively low lipid accumulation of Q26; therefore, the effect of overexpressing *TGL3* and *TGL4* was unnoticeable.

Utilization of protein engineering, peroxisome compartmentalization, and metabolic engineering, the β-farnesene titer and yield of Q26 strain reached 3.34 g/L and 0.16 g/g oleic acid, respectively, which were 221.7 and 8.3-fold higher than the initial strain, respectively. In addition, when using glucose as the carbon source, the β-farnesene yield and titer of Q26 were only 2.16 g/L and 0.031 g/g glucose, demonstrating the potential of using lipids as the feedstock for the production of acetyl-CoA-derived chemicals by engineered *Y. lipolytica*.

### β-Farnesene fermentation from oleic acid and waste lipids in bioreactors

**Fig. 6:**
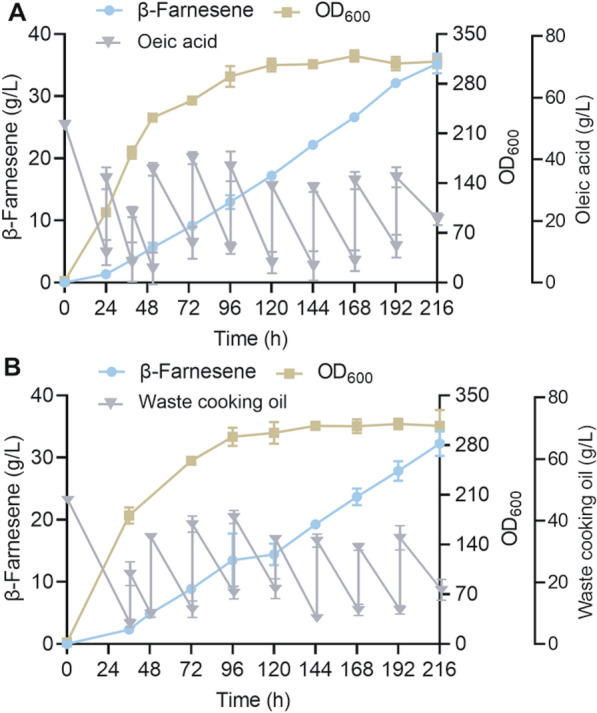
β-Farnesene production, substrate consumption, and cell growth of strain Q26 in fed-batch cultivation. The cultivation was conducted in a 5 L bioreactor at pH 6.0, 1.5 vvm airflow, and a 700 rpm stirring rate. Oleic acid (**A**) and waste cooking oil (**B**) were used as the feedstocks. Data represent the mean ± SD of two biological replicates

Finally, fed-batch cultivation was performed using Q26 to explore its potential for β-farnesene production. After optimization and comparison of the fermentation conditions (Additional file 1: Figure S5), we found that the optimal conditions for an air flux and stirring rate, pH of fermentation broth, and initial oleic acid concentration were 1.5/700 vvm/rpm, 6.5, and 50 g/L, respectively. Sterile oleic acid was also added during fermentation when the remaining content in the medium was not sufficient to be maintained for 12 h. After a 216 h cultivation (Fig. 6A), the accumulation of β-farnesene reached 35.2 g/L, and the yield was 0.17 g/g oleic acid, which was the highest reported titer for β-farnesene production in *Y. lipolytica* to date. The OD_600_ value reached 310 at the end of fermentation. The total oleic acid amount that was added to fed-batch fermentation is 209.5 g/L. In addition, there was 2.9 g/L mevalonate, 4.9 g/L mannitol, and 21.3 g/L citric acid in the fermentation broth (Additional file 1: Table S4). Strategies to reduce the accumulation of these by-products should be investigated in subsequent studies. The accumulations of lipids and squalene were 102.9 and 1.71 mg/g DCW, respectively. The accumulations of these by-products were lower than that when using glucose as the carbon source [12, 13].

We also explored the β-farnesene synthesis ability of the Q26 strain using WCO as the substrate. WCO is usually generated from the catering industry, and the improper handling of WCO can cause environmental problems. However, WCO is an ideal substrate for fermentation to produce value-added products. Here, we found that the β-farnesene production reached 31.9 g/L when using WCO as the carbon source with total amounts of 245.0 g/L within fed-batch fermentation (Fig. 6B). The result shows that the Q26 strain has great potential for the sustainable biotechnological production of β-farnesene from waste lipids.

## Conclusions

This study demonstrated the potential of *Y. lipolytica* to produce β-farnesene using lipid as the substrate. The final engineered strain produced 35.2 g/L β-farnesene using oleic acid, and 31.9 g/L β-farnesene using WCO, which was the highest titer observed for *Y. lipolytica*. This work indicated that the combination of peroxisome subcellular engineering and protein engineering with metabolic engineering can effectively boost the production of β-farnesene, and could be used as a reference for the synthesis of other terpenoids from lipid feedstocks; thereby, contributing to the reutilization of WCO.

## Material and methods

### Strains, media, and culture conditions

*E. coli* DH5α cells were used for plasmid construction and proliferation and BL21 (DE3) cells for protein purification. *E. coli* cells were cultured at 37 °C in Luria–Bertani (LB) medium (10 g/L NaCl, 5 g/L yeast extract, 10 g/L tryptone) with 20 g/L of agar added for the preparation of solid medium. Kanamycin (50 mg/L) or ampicillin (50 mg/L) was added to the LB medium when necessary. *Y. lipolytica* strains were cultured at 30 °C in YPD medium (20 g/L tryptone, 20 g/L glucose, 10 g/L yeast extract). Synthetic dextrose (SD) medium [20 g/L glucose, 1.7 g/L yeast nitrogen base, 5 g/L (NH_4_)_2_SO_4_] supplemented with suitable amino acid dropout mixes was used for the selection of transformants. Hygromycin B (0.4 g/L; Yeasen, Shanghai, China), nourseothricin (0.5 g/L; Sigma, Shanghai, China), bleomycin (0.8 g/L; Solarbio, Beijing, China), and 20 g/L agar were added to the YPD or SD medium when necessary. The strains of *Y. lipolytica* used in this study are listed in Table 1.

**Table 1:** Strains used in this study

Name	Description	Reference
PO1f	*MatA*, *Leu2-270*, *URA3-302*, *xpr2-322*, *axp-2*	INRA
AHH12	PO1f harboring linearized plasmid pki-AtoB-HMGR-HMGS	[12]
CP7	AHH12 harboring linearized plasmid pki-AtoB-HMGR-HMGS-ePTS1	This study
Q3	CP7 harboring linearized plasmid YLEP-Leu-ERG20Aan	This study
Q4	CP7 harboring linearized plasmid YLEP-Leu-ERG20mAan	This study
Q5	Q4 harboring linearized plasmid pki-ERG12-IDI-ERG20mAan	This study
Q6	Q5 harboring linearized plasmids pki-ERG12-IDI-ERG20mAan and 114-GPPS-ERG8-ERG19	This study
Q7	Linearized plasmid pki-ERG12-IDI-ERG20mAan randomly integrated into the genome of Q6 again	This study
Q8	Overexpressing *FAA1* in Q7 strain	This study
Q9	Overexpressing *PXA1* and *Pxa2* in Q7 strain	This study
Q10	Overexpressing *POX2* and *POX3* in Q7 strain	This study
Q11	Overexpressing *MFE1* in Q7 strain	This study
Q12	Overexpressing *POT1* in Q7 strain	This study
Q13	Overexpressing *PAT1* in Q7 strain	This study
Q14	Overexpressing *PEX10* in Q7 strain	This study
Q15	Overexpressing *AMPD* and *YHM2* in Q7 strain	This study
Q16	Overexpressing *ACL1* and *ACL2* in Q15 strain	This study
Q17	Overexpressing *PXA1* and *PXA2* in Q12 strain	This study
Q18	Overexpressing *POX2* and *POX3* in Q12 strain	This study
Q19	Overexpressing *MFE1* in Q12 strain	This study
Q20	Overexpressing *PAT1* in Q12 strain	This study
Q21	Overexpressing *PEX10* in Q12 strain	This study
Q22	Overexpressing *AMPD* and *YHM2* in Q12 strain	This study
Q23	Overexpressing *TGL3* and *TGL4* in Q12 strain	This study
Q24	The deletion of *DGA1* in Q12 strain	This study
Q25	The deletion of *DGA2* in Q12 strain	This study
Q26	The deletion of *DGA1* and *DGA2* in Q12 strain	This study
Q27	Overexpressing *TGL3* and *TGL4* in Q26 strain	This study

### Plasmids and strains construction

The primers used in this study were synthesized by Personalbio (Qingdao, China) and are listed in Table S1. DNA fragments were obtained by polymerase chain reaction using DNA polymerase (Vazyme, Nanjing, China). The restriction enzymes used in this study were purchased from Thermo Fisher Scientific (Shanghai, China). Finally, Gibson assembly [39] was used for plasmid construction. The plasmids used in this study are listed in Table 2. The overview of strains construction is described in Additional file 1: Figure S6.

**Table 2:** Primary plasmids used in this study

Name	Description	Reference
YLEP-Leu/nat	*Y. lipolytica* episomal vector, *ut8* promoter with *CYC1* terminator, *Leu2* or nourseothricin selection marker	[47]
pki-2/nat	*Y. lipolytica* integrative vector, *hp4d* promoter with *XPR2* terminator, *TEF* promoter with *CYC1* terminator, *URA3* or nourseothricin selection marker	[12]
YLEP-Leu-Aan	YLEP-Leu vector containing codon-optimized *AanFS* under *ut8* promoter	This study
YLEP-Leu-Him	YLEP-Leu vector containing codon-optimized *HimFS* under *ut8* promoter	This study
YLEP-Leu-Mas	YLEP-Leu vector containing codon-optimized *MasFS* under *ut8* promoter	This study
YLEP-Leu-Pme	YLEP-Leu vector containing codon-optimized *PmeFS* under *ut8* promoter	This study
pET28a-Aan	pET28a vector containing codon-optimized *AanFS* under *T7* promoter	This study
pET28-mAan	pET28a vector containing codon-optimized *AanFS*^*K197T/F180H*^ under *T7* promoter	This study
YLEP-Leu-Aan-ERG20	YLEP-Leu vector containing *AanFS* under *ut8* promoter and *GPD*-*ERG20*-*XPR2* fragments	This study
YLEP-Leu-AanERG20	YLEP-Leu vector containing fusion gene of codon-optimized *AanFS* and *ERG20* (*AanFSERG20*) with GGGGS linker and *ERG20* is at the 3' end	This study
YLEP-Leu-ERG20Aan	YLEP-Leu vector containing fusion gene of codon-optimized *AanFS* and *ERG20* (*ERG20AanFS*) with GGGGS linker and *AanFS* is at the 3' end	This study
YLEP-Leu-ERG20mAan	YLEP-Leu-ERG20Aan vector with the K197T/F180H mutation in *AanFS*	This study
pki-ERG12-IDI-ERG20mAan	pki-2 vector containing *ERG20FS*^*K197T/F180H*^ under *hp4d* promoter, *ERG12* under *ut8* promoter and *TEF*-*IDI*-*CYC1* fragments	This study
114-GPPS-ERG8-ERG19	114-EXP-FBA vector containing *GPPS* under *EXP* promoter, *GPD*-*ERG8*-*XPR2* and *TEF*-*ERG19*-*CYC1* fragments	This study
pki-AtoB-HMGR-HMGS	pKi-2 vector containing codon-optimized *AtoB* under *TEF* promoter, *HMGS* under *hp4d* promoter, and *ut8*-*HMGR*-*CYC1* fragments	This study
pki-AtoB-HMGR-HMGS-ePTS1	The ePTS1 peroxisome localization signal was fused at *C*-terminal of *AtoB*, *HMGR* and *HMGS* separately of pki-AtoB-HMGR-HMGS plasmid	This study
YLEP-nat-FAA1	YLEP-nat vector containing *FAA1* gene under *ut8* promoter	This study
YLEP-nat-MFE2	YLEP-nat vector containing *MFE1* gene under *ut8* promoter	This study
YLEP-nat-PAT1	YLEP-nat vector containing *PAT1* gene under *ut8* promoter	This study
YLEP-nat-PEX10	YLEP-nat vector containing *PEX10* gene under *ut8* promoter	This study
YLEP-nat-POT1	YLEP-nat vector containing *POT1* gene under *ut8* promoter	This study
pki-nat-PXA1/2	pki-nat vector containing *PXA1* and *PXA2* genes	This study
pki-nat-TGL3/4	pki-nat vector containing *TGL3* and *TGL4* genes	This study
pki-nat-POX2/3	pki-nat vector containing *POX2* and *POX3* genes	This study
pki-nat-YHM2-AMPD	pki-nat vector containing *AMPD* and *YHM2* genes	This study
pki-Bleor-ACL1/2	pki-Bleor vector containing *ACL1* and *ACL2* genes with bleomycin selection marker	This study
pCAS1yl-gDGA1	pCAS1yl plasmid with gRNA of *DGA1* and bleomycin selection marker	This study
pCAS1yl-gDGA2	pCAS1yl plasmid with gRNA of *DGA2* and bleomycin selection marker	This study
pCAS1yl-gDGA2	pCAS1yl plasmid with gRNA of *DGA2* and bleomycin selection marker	This study

To locate the synthesis pathway of mevalonate in the peroxisome, plasmid pki-AtoB-HMGR-HMGS-ePTS1 was constructed based on vector pki-AtoB-HMGR-HMGS by fusing the ePTS1 single coding sequence [15] to the *C*-terminus of *AtoB*, *HMGR*, and *HMGS*. The linear plasmid was transformed to AHH12 to generate CP7 strain. The β-farnesene synthase coding sequences from different plants were optimized following the codon preference of *Y. lipolytica* (listed in Additional file 1: Table S2) and inserted in the YLEP-Leu plasmid. The genes were controlled by a synthetic strong promoter *ut8*, which contains eight repeated upstream activation sequence (UAS) of alkaline extracellular protease promoter [40]. A series of plasmids containing different individual or combined mutations constructed using the YLEP-Leu-AanFS plasmid were used to identify the effects of the mutations of different amino acid residues on the β-farnesene synthase activity. To purify AanFS and AanFS^K197T/F180H^, pET28-AanFS and pET28-AanFS^K197T/F180H^ were constructed. Plasmids pki-ERG12-IDI-ERG20mAan, 114-GPPS-ERG8-ERG19 and YLEP-Leu-ERG20mAan were constructed to overexpress genes of MVA pathway, and Q3–Q7 strains were obtained by transforming these linear plasmids into CP7 strains. Genes involved in β-oxidation, *FAA1*, *PXA1*, *PXA2*, *POX2*, *POX3*, *MFE1*, *POT1*, *PAT1*, and *PEX10*, were used to construct the YLEP-nat plasmid under the control of the *ut8* promoter and *CYC1* terminator. These plasmids were transformed in Q7 strain, respectively, to obtain Q8-Q14 strains. *AMPD*, *YHM2*, *ACL1*, *ACL2*, *TGL3* and *TGL4* were also constructed in pki-nat and pki-Bleor plasmids under the control of *hp4d* and *TEF* promoters, and *XPR2* and *CYC1* terminators, respectively. *AMPD* and *YHM2* were overexpressed in Q7 strain to obtain Q15 strain. *ACL1* and *ACL2* were overexpressed in Q15 strain to obtain Q16 strain. Q23 strain was obtained by overexpressing *TGL3* and *TGL4* in Q12 strain. The CRISPR–Cas9 system was used to delete *DGA1* and *DGA2* to obtain Q24–Q26 strains. The sgRNA was designed to create double strand break (DSB), and DSB was repaired by non-homologous end joining to introduce nucleotide deletion or insertion. The deletion of *DGA1/2* genes was confirmed by PCR of the genomic DNA and sequencing. Detailed information on the aforementioned genes is listed in Additional file 1: Table S3. To obtain the gene overexpression strain, the plasmids were linearized by digestion with a restriction enzyme and transformed into *Y. lipolytica* for random genome integration. After transformation, the transformants were picked and colonies PCR were performed to verify that the targeted genes were integrated into the genome. A total of 20 transformants were selected, and the production of β-farnesene was determined by shake-flask fermentation. The transformants with relatively high β-farnesene production were further engineered. Finally, DNA fragments were transformed into *Y. lipolytica* strains using the lithium acetate method [41].

### Screening of farnesene synthase

To screen β-farnesene synthase with higher activity in *Y. lipolytica*, β-farnesene synthases from different plants were used. The evolutionary tree of β-farnesene synthase was constructed using MEGA 5.0, and β-farnesene synthases distributed in different branches of the evolutionary tree were selected. The corresponding DNA coding sequences were synthesized and constructed into the expression plasmid of *Y. lipolytica* according to its codon preference*.* The β-farnesene titers of strains with the different β-farnesene synthases were compared after fermentation, and the source with the highest titer was selected as the target.

### Fluorescence microscopy

Strains containing the corresponding plasmids were grown in YPD medium overnight for the verification of the localization efficiency of the ePTS1 signal peptide. Cultures were concentrated and washed with phosphate-buffered saline. Cell suspensions were spotted onto plain glass slides and examined using a research-grade inverted fluorescence microscope (NIKON TI-E, Japan) with 100× objective, and 485 and 587 nm excitation lasers for GFP and mCherry, respectively.

### Analysis of oleic acid and intracellular lipids

To determine the oleic acid content in the culture medium, 1 mL of fermentation broth was taken and centrifuged to remove the cells, and *n*-hexane (400 μL) was added to extract the oleic acid. Sulfuric acid/methanol (2:1, 1 mL) was added and the mixture was incubated at 60 °C for 2 h after the *n*-hexane was volatilized for the methylation of oleic acid. Dodecane (200 μL) was added to extract the methyl oleate, and then the content of methyl oleate was quantitatively analyzed by gas chromatography (GC). To measure the content of intracellular lipids, 1 mg of cells was collected, washed with sterile water three times, and resuspended in 1 mL of chloroform/methanol (2:1) for 1 h. Normal saline (125 μL) was added to the organic phase and the mixture was thoroughly vibrated after centrifugation. The organic phase was collected and evaporated in the oven, and the obtained lipids were suspended in 100 μL of *n*-hexane for methylation. Finally, fatty acid methyl esters were extracted with 200 μL of dodecane for quantitative analysis. The analysis program of the GC system with a flame ionization detector (FID) and Rtx-5 capillary column (30.0 m, 0.25 mm ID, 0.25 μm df; RESTEK, USA) was as follows: the temperatures of the injector and FID were set to 250 and 280 °C, respectively, the oven temperature was maintained at 80 °C for 1 min, increased to 250 °C at 20 °C/min, and then held for 15 min.

### Analysis of OD_600_, mevalonate, citric acid, and mannitol

The biomass of the fermentation broth was determined using a UV-1800 spectrophotometer (Shimadzu, Japan) at 600 nm after washing and diluting with sterile water. To analyze the accumulation of mevalonate, mannitol, and citric acid during fermentation, a sample of fermentation broth (1 mL) was taken and centrifuged to remove cell debris. Next, the supernatant was treated twice with 200 μL of *n*-hexane to remove the remaining lipids and then filtered through a 0.22 μm filter. A high-performance liquid chromatography (HPLC) system equipped with an Aminex HPX-87H column (BioRad, Inc., Hercules, CA, USA) and a refractive index detector was used to analyze mevalonate, citrate, and mannitol. H_2_SO_4_ (5 mM) was used as the mobile phase with a flow rate of 0.6 mL/min at 65 °C.

### Analysis of β‑farnesene

β-Farnesene was captured by dodecane during fermentation. The dodecane was collected and centrifuged to eliminate cell debris, then injected into a GC system equipped with a FID and Rtx-5 capillary column. The detection program was as follows: the injector and the detector temperatures were set to 280 and 290 °C, respectively. The oven temperature was maintained at 80 °C for 1 min, increased to 250 °C at 10 °C/min and held for 1 min, increased to 280 °C at 10 °C/min and held for 2 min.

### Fed-batch fermentation

The fermentation medium was 2 ×YP with 50 g/L oleic acid or WCO. The working volume of the 5 L bioreactor (Eppendorf NBS BioFlo 310, USA) was 4 L. The fermentation broth pH values investigated were 5.5, 6.0 or 6.5, and the air flux and stirring rate were set to 1.5/600, 1.5/700, or 2.0/700 (vvm/rpm). During the fermentation seed preparation, the Q26 strain was inoculated into a test tube containing 5 mL of YPD medium and cultured at 30 °C and 220 rpm for 24 h. The culture (50 μL) was inoculated into a 300-mL flask containing 50 mL of YPD medium and cultured under the same conditions. The 300-μL second pre-culture was transferred into a 1000-mL flask containing 300 mL of YPD medium for culturing for the final fermentation seeds. The prepared culture was transferred to a bioreactor with 10% dodecane. Sterile oleic acid or WCO was added during fermentation when the remaining content in the medium was insufficient to be maintained for 12 h.

### Model prediction and mutation site calculation for β-farnesene synthase

Homologous modeling was used to create a model of the three-dimensional structure of β-farnesene synthase. We used multitemplate modeling because of the poor quality of the templates. The PDB identifiers of the three protein structure templates were 4GAX (coverage: 0.98; identity: 0.49), 4FJQ (coverage: 0.98; identity: 0.50), and 5H7T (coverage: 0.96; identity: 0.43). Modeller 9.12 was used for the homologous modeling of β-farnesene synthase using the three protein structure templates above.

The molecular dynamics (MD) simulation used NAMD 2.12 [42] with the CHARMM22 [43, 44] force field. The simulations were conducted using periodic boundary conditions, including a 12 Å cutoff for the nonbonded interactions and particle-mesh Ewald for the long-range electrostatic interactions. A time step of 2 fs was used, and snapshots were saved every 1 ps. The protein was fully constrained, and the solvent was minimized for 2000 steps using a conjugate gradient algorithm. The solvent was equilibrated for 100 ps under constant temperature and pressure NPT conditions. The solvent was fully constrained, and the protein was minimized for 2000 steps. The entire system was minimized for another 2000 steps and equilibrated for 100 ps under the same NPT conditions. Finally, 20 ns MD simulation at 303 K in constant energy and constant-volume ensemble NVE conditions was performed [45].

To obtain variants with improved catalytic activity, HotSpot Wizard 3.0 [46] was used to design β-farnesene synthase variants. Because of the poor quality of the homologous modeling structures, MD simulations were employed for the structural optimization of β-farnesene synthase. Concurrently, in considering the complexity of the protein conformation, we uniformly sampled the MD simulation trajectory of β-farnesene synthase. We denoted the amino acid residues in the product-release region of β-farnesene synthase as the second optimized target. According to the analysis results of the MD simulation trajectory of the β-farnesene synthase–farnesene structure, the main amino acid residues influencing the release of β-farnesene during the farnesene synthesis were predicted. Finally, these amino acid residues were replaced with amino acids with different side-chain types.

## Supplementary Information

**Additional file 1: Figure S1.** Screening β-farnesene synthase from different plants. **A** The evolutionary tree of β-farnesene synthase from different plants. **B** β-Farnesene production and biomass of strains containing different β-farnesene synthase. Data represent the mean ± SD of biological triplicate. **Figure S2.** The SDS-PAGE electrophoresis analysis of purified protein AanFS^K197T/F180H^ and AanFS. **Figure S3.** The copy number and relative expression of *AanFS*^*K197T/F180H*^ in Q6 and Q7 strains. Data represent the mean ± SD of biological duplicate. **Figure S4.** Intracellular lipids accumulation in PO1f, CP7 and Q12 strain. Data represent the mean ± SD of biological triplicate. **Figure S5.** Fermentation conditions optimization of Q26 strain in 5 L bioreactor. **A** β-Farnesene production at different air flux and stirring rate. **B** Growth curve at different air flux and stirring rate during fermentation. C β-Farnesene production at different pH. D Growth curve at different pH. β-Farnesene detected after 96 h of fermentation. Data represent the mean ± SD of biological duplicate. **Figure S6.** The relationship of the engineered strains used in this study. CP7 strain was obtained by locating the synthesis pathway of mevalonate in the peroxisome of AHH12 strain. Different genes of MVA pathway were successively overexpressed in CP7 strain to obtain Q3–Q7 strains, respectively. On the basis of strain Q7, the genes involved in β-oxidation were overexpressed, respectively, to construct strain Q8–Q14, and Q17–Q21 strain was obtained by overexpressing other genes related to β-oxidation basing on Q12 strain. Q15 strain was constructed by regulating citric acid metabolism of Q7 strain and Q16 strain was derived from Q15 strain. Expressing citric acid regulating genes in Q12 strain obtained Q22 strain. On the basis of strain Q12, the fatty acid anabolism was regulated to construct Q23–Q26 strain. On the basis of strain Q26, the combinatorial regulation of fatty acid anabolism obtained Q27 strain. **Table S1.** The primary primers used in this study. **Table S2.** The codon optimized sequences of β-farnesene synthase from different plants. **Table S3.** The detailed information of genes used in this study. **Table S4.** The accumulation of by-products at 216 h of fermentation. Data represent the mean ± SD of biological duplicate.

## Data Availability

The datasets used and analyzed during the current study are available from the corresponding author on reasonable request.

## Abbreviations

MVA: Mevalonate
IPP: Isopentenyl diphosphate
FPP: Farnesyl pyrophosphate
AtoB: Acetyl-CoA acetyltransferase
HMGS: HMG-CoA synthase
HMGR: HMG-CoA reductase
ERG12: Mevalonate kinase
ERG8: Phosphomevalonate kinase
IDI: IPP isomerase
ERG20: FPP synthase
ERG19: Mevalonate diphosphate decarboxylase
AanFS: β-Farnesene synthase from *A. annua*
TAGs: Triacylglycerides
DAGs: Diacylglycerols
