# Supplementary material for: Engineering Yarrowia lipolytica for the sustainable production of β-farnesene from waste oil feedstock
Source: Biotechnol Biofuels Bioprod. 2022 Oct 3;15:101. doi: 10.1186/s13068-022-02201-2 (PMC9528160; doi:10.1186/s13068-022-02201-2)
Supplement: Supplementary file 1 — Additional file 1: Figure S1. Screening β-farnesene synthase from different plants. A The evolutionary tree of β-farnesene synthase from different plants. B β-Farnesene production and biomass of strains containing different β-farnesene synthase. Data represent the mean ± SD of biological triplicate. Figure S2. The SDS-PAGE electrophoresis analysis of purified protein AanFSK197T/F180H and AanFS. Figure S3. The copy number and relative expression of AanFSK197T/F180H in Q6 and Q7 strains. Data represent the mean ± SD of biological duplicate. Figure S4. Intracellular lipids accumulation in PO1f, CP7 and Q12 strain. Data represent the mean ± SD of biological triplicate. Figure S5. Fermentation conditions optimization of Q26 strain in 5 L bioreactor. A β-Farnesene production at different air flux and stirring rate. B Growth curve at different air flux and stirring rate during fermentation. C β-Farnesene production at different pH. D Growth curve at different pH. β-Farnesene detected after 96 h of fermentation. Data represent the mean ± SD of biological duplicate. Figure S6. The relationship of the engineered strains used in this study. CP7 strain was obtained by locating the synthesis pathway of mevalonate in the peroxisome of AHH12 strain. Different genes of MVA pathway were successively overexpressed in CP7 strain to obtain Q3–Q7 strains, respectively. On the basis of strain Q7, the genes involved in β-oxidation were overexpressed, respectively, to construct strain Q8–Q14, and Q17–Q21 strain was obtained by overexpressing other genes related to β-oxidation basing on Q12 strain. Q15 strain was constructed by regulating citric acid metabolism of Q7 strain and Q16 strain was derived from Q15 strain. Expressing citric acid regulating genes in Q12 strain obtained Q22 strain. On the basis of strain Q12, the fatty acid anabolism was regulated to construct Q23–Q26 strain. On the basis of strain Q26, the combinatorial regulation of fatty acid anabolism obtained Q27 strain. Table [file 13068_2022_2201_MOESM1_ESM.docx]

**Additional File Information**

**Engineering *Yarrowia lipolytica* for the sustainable production of β-farnesene from waste oil feedstock**

Yinghang Liu^#^, Jin Zhang^#^, Qingbin Li, Zhaoxuan Wang, Zhiyong Cui, Tianyuan Su, Xuemei Lu, Qingsheng Qi*, Jin Hou*

State Key Laboratory of Microbial Technology, Shandong University, Qingdao 266237, China

* Corresponding author: Prof. Jin Hou, email: [houjin@sdu.edu.cn](mailto:houjin@sdu.edu.cn), Prof. Qingsheng Qi, email: [qiqingsheng@sdu.edu.cn](mailto:qiqingsheng@sdu.edu.cn), State Key Laboratory of Microbial Technology, Shandong University, Binhai Road 72, Qingdao 266237, China, telephone: +86 53258632401

^#^ To be considered as joint first authors.


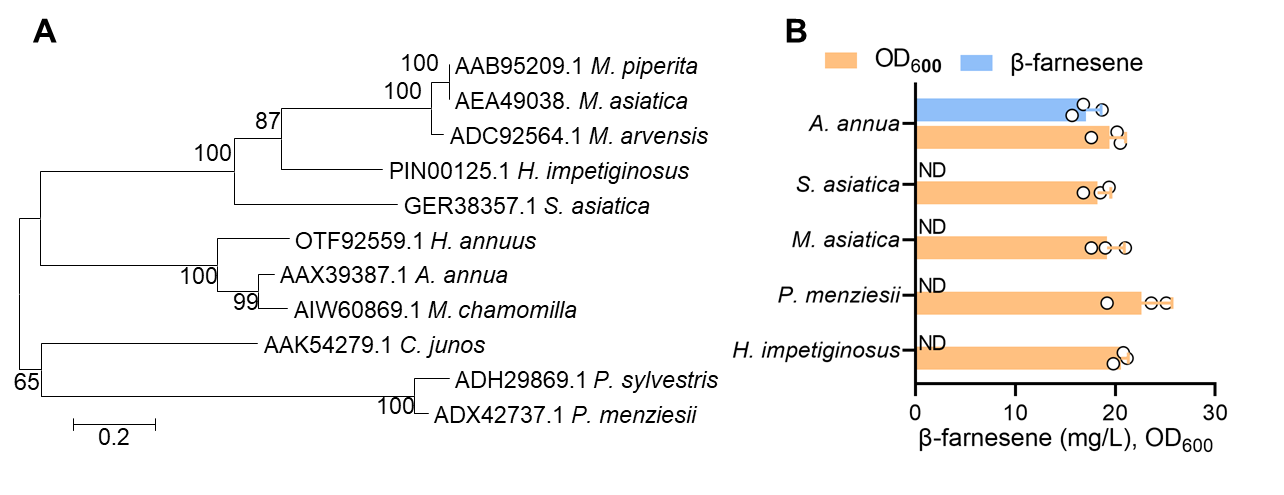


**Additional file 1: Figure S1.** Screening β-farnesene synthase from different plants. (A) The evolutionary tree of β-farnesene synthase from different plants. (B) β-Farnesene production and biomass of strains containing different β-farnesene synthase. Data represent the mean ± SD of biological triplicate.


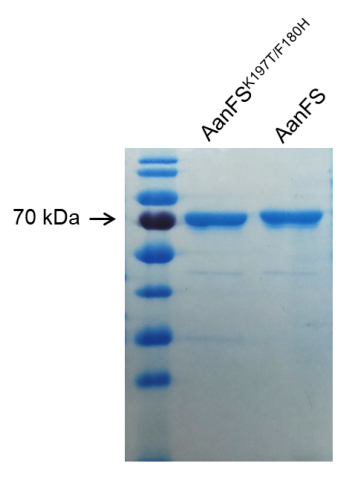


**Additional file 1: Figure S2.** The SDS-PAGE electrophoresis analysis of purified protein AanFS^K197T/F180H^ and AanFS.


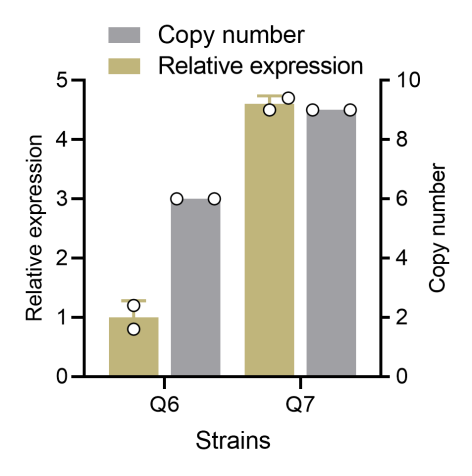


**Additional file 1: Figure S3.** The copy number and relative expression of *AanFS^K197T/F180H^* in Q6 and Q7 strains. Data represent the mean ± SD of biological duplicate.


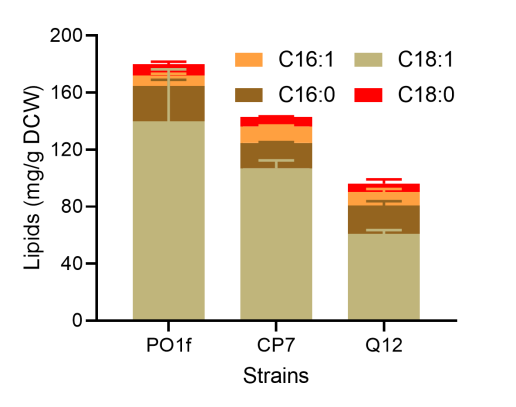


**Additional file 1: Figure S4.** Intracellular lipids accumulation in PO1f, CP7 and Q12 strain. Data represent the mean ± SD of biological triplicate.


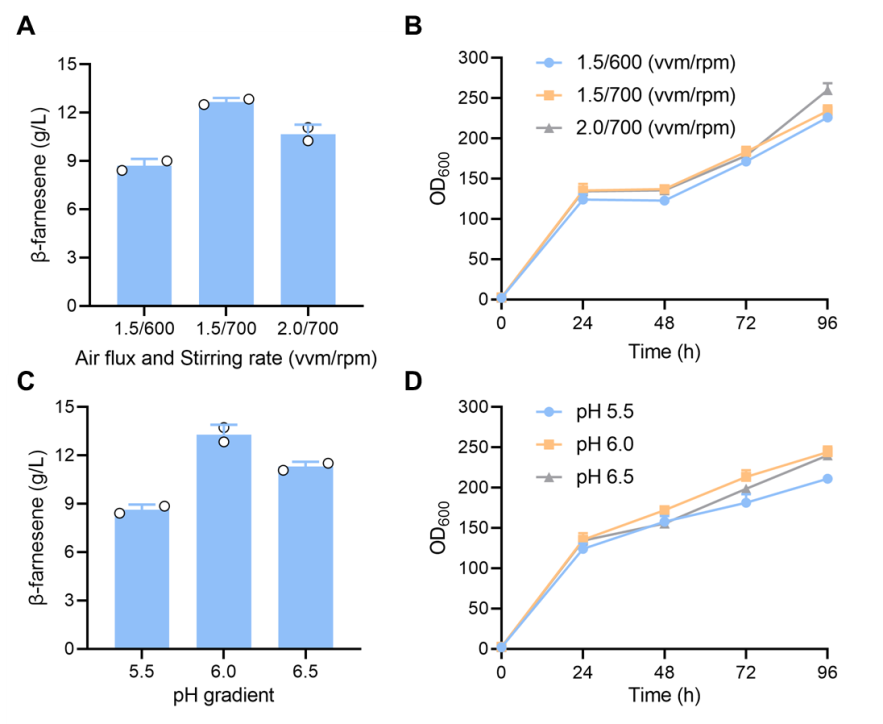


**Additional file 1: Figure S5.** Fermentation conditions optimization of Q26 strain in 5-L bioreactor. (A) β-Farnesene production at different air flux and stirring rate. (B) Growth curve at different air flux and stirring rate during fermentation. (C) β-Farnesene production at different pH. (D) Growth curve at different pH. β-Farnesene detected after 96 h of fermentation. Data represent the mean ± SD of biological duplicate.


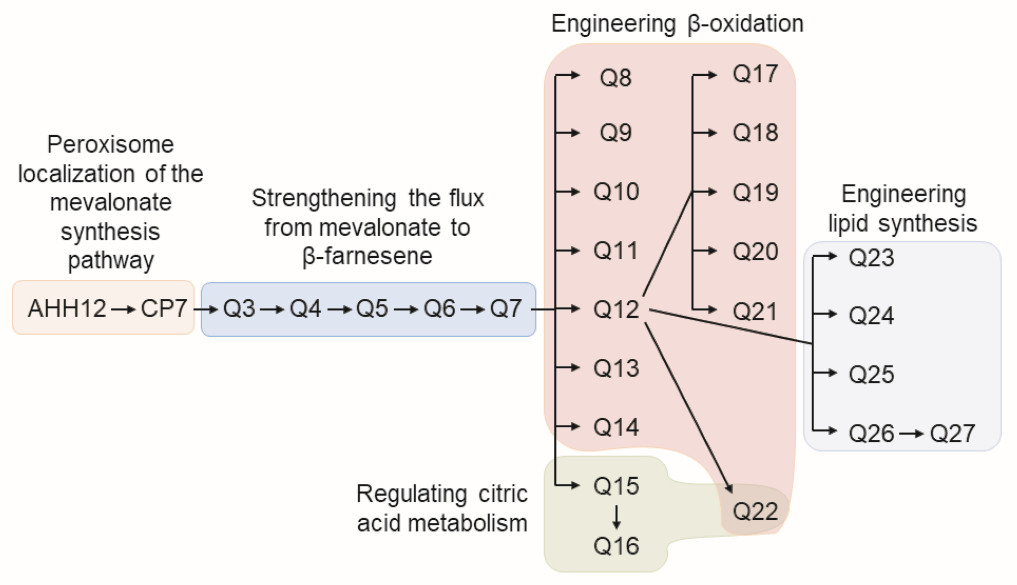


**Additional file 1: Figure S6.** The relationship of the engineered strains used in this study. CP7 strain was obtained by locating the synthesis pathway of mevalonate in the peroxisome of AHH12 strain. Different genes of MVA pathway were successively overexpressed in CP7 strain to obtain Q3-Q7 strains, respectively. On the basis of strain Q7, the genes involved in β-oxidation were overexpressed respectively to construct strain Q8-Q14, and Q17-Q21 strain was obtained by overexpressing other genes related to β-oxidation basing on Q12 strain. Q15 strain was constructed by regulating citric acid metabolism of Q7 strain and Q16 strain was derived from Q15 strain. Expressing citric acid regulating genes in Q12 strain obtained Q22 strain. On the basis of strain Q12, the fatty acid anabolism was regulated to construct Q23-Q26 strain. On the basis of strain Q26, the combinatorial regulation of fatty acid anabolism obtained Q27 strain.

**Additional file 1: Table S1** The primary primers used in this study.

| Name | Sequence (5’-3’) | Purpose |
| --- | --- | --- |
| Aan-pET-F | gacggagctcgaattcggatccttacaccaccatggggtgcac | Amplifying AanFS to pET28a |
| Aan-pET-R | cagcaaatgggtcgcggatcccatcatcaccatcaccatatgtctaccctgcctatctc |  |
| GERG20-F | ctatggaaaaacgccatatgggttgaaatgaatcggccga | Amplifying *GPD*-*ERG20*-*XPR2* fragment |
| XERG20-R | cttccttggtaccttcatatgcatctcacttgcgtatgtat |  |
| FAA1-F | ataagaatcattcaaaggttcgaaatggtcggatacacaatttc | Amplifying *FAA1* |
| FAA1-R | cataactaattacatgattcgaactaagactgctcgtagcact |  |
| MFE2-F | agaatcattcaaaggttcgaaatgtctggagaactaagata | Amplifying *MFE1* |
| MFE2-R | cataactaattacatgattcgaattagagcttagcatccttg |  |
| POX2-F | tccacgtgggaaccgcgatcgcatgaaccccaacaacactggc | Amplifying *POX2* |
| POX2-R | gccatggaggtacgcgatcgcctattcctcatcaagctcgc |  |
| POX3-F | ctaattacatgaatttaaatctattcctcgtccagctcgc | Amplifying *POX3* |
| POX3-R | gtactaaccgcagatttaaatatgatctcccccaacctcac |  |
| POT1-F | agaatcattcaaaggttcgaaatggaccgacttaacaacct | Amplifying *POT1* |
| POT1-R | cataactaattacatgattcgaattactcggcaacaaccagag |  |
| PAT1-F | gaatcattcaaaggttcgaaatgcgactcactctgccccg | Amplifying *PAT1* |
| PAT1-R | cataactaattacatgattcgaactactcgacagaagagacct |  |
| PEX10-F | agaatcattcaaaggttcgaaatgtggggaagttcacatgc | Amplifying *PEX10* |
| PEX10-R | cataactaattacatgattcgaattatctgataggcaacaag |  |
| PXA2-F | cataactaattacatgaatttaaatctactgagcaacagacgcct | Amplifying *PXA2* |
| PXA2-R | gcagtactaaccgcagatttaaatatggaccgtctcaaaacgtc |  |
| PXA1-F | cacatccacgtgggaaccgcgatcgcatggcaaacgtctctacact | Amplifying *PXA1* |
| PXA1-R | gacaggccatggaggtacgcgatcgcttatttcttctcggacttc |  |
| TGL3-F | gtactaaccgcagatttaaatatgaaaagccgcgtggccgt | Amplifying *TGL3* |
| TGL3-R | cataactaattacatgaatttaaatctagttttgtcgcttggtct |  |
| TGL4-F | cacgtgggaaccgcgatcgcatgttcacctccagagtttc | Amplifying *TGL4* |
| TGL4-R | gccatggaggtacgcgatcgcttagcacgagtcagaacagt |  |
| AMPD-F | cataactaattacatgaatttaaatttaaccatgcagccgctcaaac | Amplifying *AMPD* |
| AMPD-R | gcagtactaaccgcagatttaaatatgccgcagcaagcaatggat |  |
| YHM2-F | cacatccacgtgggaaccgcgatcgcatgggtgctgctaacctcaac | Amplifying *YHM2* |
| YHM2-R | gacaggccatggaggtacgcgatcgcctagtgcttaccaacaggtcg |  |
| ACL1-F | gcagtactaaccgcagatttaaatatgtctgccaacgagaacatc | Amplifying *ACL1* |
| ACL1-R | cataactaattacatgaatttaaatctatgatcgagtcttggccttg |  |
| ACL2-F | gcagtactaaccgcagatttaaatatgtcagcgaaatccattcac | Amplifying *ACL2* |
| ACL2-R | cataactaattacatgaatttaaatttaaactccgagaggagtggaag |  |
| gRNA-F | gttttagagctagaaatagc | Amplifying pCAS1yl-gDGA1/2 plasmid |
| dDGA1-R | gctatttctagctctaaaaccgctatcgacaccattactcgacgagcttactcgtttcg |  |
| dDGA2-R | gctatttctagctctaaaacctgagcgacagggtggacgagacgagcttactcgtttcg |  |
| AE-F | gtataagaatcattcaaaggttcgaaatgtctaccctgcctatctc | Amplifying *AanFS^K197T/F180H^ERG20* |
| AE-R | actaattacatgattcgaactacttctgtcgcttgtaaatc |  |
| EA-F | ccacgtgggaaccgcgatcgcatgtccaaggcgaaattcg | Amplifying *ERG20AanFS^K197T/F180H^* |
| EA-R | cataactaattacatgattcgaattacaccaccatggggtgc |  |

**Additional file 1: Table S2** The codon optimized sequences of β-farnesene synthase from different plants.

| Description | DNA base sequence (5’ to 3’) |
| --- | --- |
| The optimized β-farnesene synthase coding sequence from *Artemisia annua* according to the codon preference of *Y. lipolytica* | atgtctaccctgcctatctcttctgtgtctttctcgtcctctacctctcctctggtggtggacgacaaggtgtctaccaagcctgacgtgatccgacacaccatgaacttcaacgcctctatctggggcgaccagttcctgacctacgacgagcccgaggacctggtgatgaagaagcagctggtcgaggaactgaaggaagaggtgaagaaggaactgatcaccatcaagggctctaacgagcccatgcagcacgtgaagctgatcgagctgattgacgccgtgcagcgactgggaatcgcctaccacttcgaggaagagatcgaggaagctctgcagcacatccacgtcacctacggcgagcagtgggtcgacaaggaaaacctgcagtctatctccctgtggttccgactgctgcgacagcagggcttcaacgtgtcctctggcgtgttcaaggacttcatggacgagaagggcaagttcaaggaatctctgtgcaacgacgcccagggcattctggccctgtacgaggccgccttcatgcgagtcgaggacgaaaccatcctggacaacgccctcgagttcaccaaggtccacctggacatcattgctaaggacccctcttgcgactcttcgctgcgaacccagatccaccaggctctgaagcagcccctgcgacgacgactggcccgaatcgaggccctgcactacatgcccatctaccagcaagagacttctcacgacgaggtgctgctgaagctggccaagctggacttctccgtgctgcagtccatgcacaagaaggaattgtctcacatctgcaagtggtggaaggacctggacctgcagaacaagctgccctacgtgcgagatcgagtggtcgagggctacttctggatcctgtctatctactacgagccccagcacgctcgaacccgaatgttcctgatgaagacctgcatgtggctggtggtcctggacgacaccttcgacaactacggcacctacgaggaactcgagatcttcacccaggccgtggaacgatggtctatctcttgcctggacatgctgcccgagtacatgaagctcatctaccaagagctggtgaacctgcacgtcgagatggaagagtctctcgagaaggaaggcaagacctaccagattcactacgtgaaggaaatggccaaggaactcgtgcgaaactacctggttgaggcccgatggctcaaggaaggctacatgcctactctggaagagtacatgtctgtgtctatggtgaccggaacctacggcctgatgatcgcccgatcttacgtcggccgaggcgacatcgtgaccgaggacaccttcaagtgggtgtcgtcttaccctcctatcatcaaggcctcttgcgtgatcgtgcgactgatggacgacattgtgtctcacaaggaagaacaagagcgaggccacgtcgcctcttctatcgagtgctactctaaggaatctggcgcctctgaggaagaggcctgcgagtacatctctcgaaaggttgaggacgcctggaaggtgatcaaccgagagtctctgcgacccaccgctgtgccctttccactgctgatgcccgccatcaacctggctcgaatgtgtgaggtgctgtactccgtgaacgacggcttcacccacgctgagggcgacatgaagtcttatatgaagtcgttcttcgtgcaccccatggtggtgtaa |
| The optimized β-farnesene synthase coding sequence from *Handroanthus impetiginosus* according to the codon preference of *Y. lipolytica* | atggccgccacctctatcgagaacctgcgacctgccaaggctactttcgacccctctctgtggggcgataccttctctgccttctcgctggacgacaaggtgcaagagacttacgccgaggccatcgaggaactgcgaaaggaagccaagaagatgctgatggctcccaagtcctctaagctgctgatcctgatcgacaccatcgagcgactgggcctcgcctaccacttcgagaaggaaatccaagagaagctgcaagagatcctgcgagtgttcgagcgattcaccaacaaggacaagaagttcgaggaaaccctgattgacaacgtcgagggcctgctgaacctgtacgaggccgctcacatccgaatccacggcgaggacatcctggaagaggtggtggccttcaccactcaccatctgacccgagtgctgccccagctcgagtctcccattaaggacaaggtgaagcgagccctcgagcaccccatccaccgagctgtgcccatcatcgaggcccgactgtacatctctatctacgagaaggacgagtctcgagatgaggtgctgctgaagatggccaagctgaacttcaactacctgcagaacctctacaaggacgagctgtctcagctgtctgtgtggtggaagaagtttgacctgaagtccaagctgccctacgctcgaaaccgactgatcgagtgcttcctgtggggagtgtctttccactttgagccccagtactctcaggtgcgaatggccgtggccaagtctgtgaagctggtgaccatcattgacgacacctacgacaactacgccactctcgaggaagcccagctgttctctgagatcctggaacgatgcaacatggaccagaccgagcgagtccccgactggatgaagattgcctaccagttcgtgatgaccatctacgaggactacgagcgagatgccaccaagcaagaaaagcccttcgctatcccctacttcaaggaagccctgaagtggatcatgaagcgagagctgccctcgtttgaagagtactacgccaactccgtgatcacctcttgcctgttcgtgctgttcaccggacacttccccggcatgaagtccgtgaccaaggaaaccatcgactggctgctgtctgagcccaagatcgtgattaccgccgccaagaccggacggtactacaacgacctgggctcccatgagcgagagaacatcggcggcaagcagctgaccgccatggactgctacatgaaggaacacggcgtgtctaagcaagaggctgtgtctaagttcctggaactggtcgaggaaggctggaaggacctgaacaccgagtgggtgaccaagactcactctgtgcccaagcacatcctggtgcagctgctgaactacgtgcgagaggccgccgtgacctacaacaacggccaggacggcgtgactaaccctcaggtgctggtgccctacattcacgccctgttcgtcgagcccatcgtggtgtaa |
| The optimized β-farnesene synthase coding sequence from *Mentha asiatica* according to the codon preference of *Y. lipolytica* | atggccaccaacggcgtggtgatctcttgcctgcgagaggtgcgacctcctatgaccaagcacgctccctctatgtggaccgataccttctctaacttctcgctggacgacaaggaacagcagaagtgctctgagactatcgaggccctgaagcaagaggcccgaggcatgctgatggccgccaccactcctctgcagcagatgaccctgatcgacaccctcgagcgactgggcctgtctttccacttcgagactgagatcgagtacaagatcgagctgatcaacgccgccgaggacgacggcttcgacctgttcgccactgctctgcgattccgactgctgcgacagcaccagcgacatgtgtcttgcgacgtgttcgacaagttcatcgacaaggacggcaagttcgaggaatctctgtctaacaacgtcgagggcctgctgtctctgtacgaggctgcccacgtcggcttccgagaggaacgaatcctgcaagaggctgtgaacttcacccgacaccacctcgagggcgccgagctggaccagtctcctctgctgatccgagagaaggtgaagcgagccctcgagcaccctctgcaccgagacttccccatcgtgtacgcccgactgttcatctctatctacgagaaggacgactctcgagatgagctgctgctgaagctgtctaaggtcaacttcaagttcatgcagaacctgtacaaggaagaactgtctcagctgtctcgatggtggaacacctggaacctgaagtctaagctgccctacgctcgagacagagtggtcgaggcctacgtgtggggcgtgggctaccactacgagccccagtactcttacgtgcgaatgggcctcgccaagggcgtgctgatttgcggcatcatggacgacacctacgacaactacgctaccctgaacgaggcccagctgttcacccaggtgctggacaagtgggaccgagatgaggctgagcgactgcccgagtacatgaagatcgtgtaccgattcatcctgtccatctatgagaactacgagcgagatgccgccaagctgggcaagtctttcgctgctccctacttcaaggaaaccgtgaagcagctggcccgagccttcaacgaggaacaaaagtgggtgatggaacgacagctgccctcgttccaggactacgtgaagaactctgaaaagacctcttgcatctacaccatgttcgcctctatcatccccggactgaagtctgtgacccaagagactattgactggatcaagtctgagcccactctggccacctctaccgccatgatcggacgatactggaacgatacctcttcgcagctccgagagtctaagggcggcgagatgctgactgccctggacttccacatgaaggaatacggcctgaccaaggaagaggccgcctctaagtttgagggcctcgtcgaggaaacctggaaggacatcaacaaggagttcattgccaccaccaactacaacgtgggccgagagatcgctattaccttcctgaactacgcccgaatctgcgaggcctcttactctaagaccgacggcgacgcctactctgaccccaacgtggccaaggccaacgtggtggccctgttcgtggacgccatcgtgttctaa |
| The optimized β-farnesene synthase coding sequence from *Pseudotsuga menziesii* according to the codon preference of *Y. lipolytica* | atgtctctggaagagttctaccccatggccaccgtgtctgtgccctctactctgccctgcgctctgtctacctcttctaactcttcttctctggtgcgacgaactgctaaccctcatcctaacgtgtgggactaccacttcgtgcagtccctgcagtctccctacactgacccctgctacggcgagcgagtcgagactctggtggccgagatcaaggccatgctgcacggcgagggctgctctatgatcaccccttctgcctacgacaccgcctgggtcgctcgagtgccctccatcgacggctctgcccgacctcagttccctcagaccgtcgagtggatcctgaagaaccagctgaaggacggctcttggggaaccgagtctcacttcctgctgtctgaccgactgcctgccactctgtcttgcgtgctggccctgctgaagtggaaggtgggcgacctccaggtgctgcagggcatcgagttcatcaagtctaccctcgaggccatcaaggacgagaacgaccaggactctctggtgaccgacttcgacatcatcttcccatctctgctgcgagaggcccagtacctggacatcgagctgccctacaacctgccttacgtgtccctgctgcacaccaagcgacaagagcgactggccaacatgtctcgagaagagatccacggcgtgccctcgcctctgctgtactctctcgagggaatcgaggacatggtggactgggagcgaatcatggacgtgcgatctcaggacggatctttcctgtcctctcctgcctctaccgcctgcgtgttcatgcacaccggcgatatcaagtgcctcgagttcctgaacaacgtcctggccaacttcggcaccttcgtgccctgtctgtaccccgtggacctgctggaacgactgctgatcgtggacaacctggtgcagctgggaatcgaccgacacttcgagaaggaaatcaaggaagccctggactacgtccaccgacactggaacgagcgaggcatcggctggggccgactgaaccccattgccgacctcgagatcaccgctctgggcttccgactgctgcgactgcaccgatacaacgtgtctcccgccgtgttcgagaacttcaaggactctaacggacacttcgtgtgctctgccgctcagttcaacaaggacgtggcctctatgctgtctctgtaccgagcctctcagctggctttccccggcgagaacatcctggacgaggccaagtctttcacctctaagtacctgaaggaagctcttgagaagcgagagacttactctgcctggaacaacaagcagtctctgtctgaagagatcaagtacgccctcgagaactcttggcacgcctctgtgccccgagtcgaggctaagcgatactgccaggtgtaccgatctgactacacctacctggccaagtccgtgtacaagctgcccaaggtgaacaacgagaagatcctcgagctggccaagctggacttcaacattatccaggccatccaccaaaaggaaatgaagaacgtcaccacctggttcaagaactctgagttccctctgctgcccttcggccgagagcgacccgttgagtgcttcttcatcgtggccgctggcacctacgagccccagtacgccaagtgccgattcctgttctctaaggtggcctgcctgaacaccgtgctggacgacatgtacgacacctacggcaccctcgacgagctgaagctgttcaccgaggccgtgcgacgatgggacctgtcgctgaccgagaacctgcctgactacatgaagctgtgctacaagatcttctacgacattgtgcacgaggtggtgctcgaggctgagaaggaacagggacgagagctgctgaccttcttcagaaagggctgggaagagtacctgatgggctactacgaagaggccgagtggctggcctgcgagtacatgccctcgcttgaagagtacatccgaaacggcatcatctctatcggccagcgaatcctggtggtgtccggcgtgctgctgatggaaggccagatcctgtctcaagaggccctcgagcagctggactaccccggacgacgagtcctgaccgagctgaactccatcatcacccgactggccgacgacatccacacctacaaggccgagaaggcccgaggcgagctggcttcttctatcgagtgttacatgaaggaacaccccggctctaccgaagaggtggccgtgaactacatgtactccctgctcgagcccgccgtgaaggaactgacctgggagtttctgaagcccgaggactctaccgtgcacatcccattccagtgcaagaagatgctcatggaagagactcgagtgaccatggtgatcttcaaggaaggcgacggcttcggcatctctaagaccaagatcaaggactacattaaggactgcctgatcgagcccctgcctctgtaa |
| The optimized β-farnesene synthase coding sequence from *Striga asiatica* according to the codon preference of *Y. lipolytica* | atggccgctgccggcatgttcgaccctccatcttcttggcccaacggccgacctcctatcggcttctgcaccaagtctaagtggggcgacaccttctcttcgttcgccctggaccaccaggtgcaagagaagtacgccgaggccattgccgagctgaaggaagaggcccgaggcatgctgatccaggccgagggcaagaccatctctgagcgactccagctgatcgacaccctggaacgactcggcgtgggctaccacttcgagcaagagatcgaggaacagctgcgagacatcctgaccgagttcgactctgagcacgaggactacgacctgttcaccaccgctctgtggttccgactgctgcgacagcacggctcttacgtgtcttgcgacttcttcgacaagttcatcgagaaggaaaagaagatttctgagaacatcgagaacgacgccaagggcctgctgtctctgtacgaggccgctcatctgcgaacccacggcgaggaaatcctggacgaggccgtgaccttcaccgctcgacacctccgacgaatggtgcacgacttggagcccgctctccaggctcaggtgaagcgagcccttgagcgacccctgcaccgaggcatccagcgaatggaaacccgacactacatctggtcttacgagaaggacgactctcgaaacgagcagctcctgaagctggccaagctggacttcaactacgtgcagaacatctacaaggacgagcttggacaggtgacccgagtggtcgaggcctacctgtggggcacctctcaccactttgagccccagtactcctacgtgcgagtggccttcgccaagtacatccagatgctgaccgtgctggacgacacctacgacaactacgccaccgtggaagagggcgacatcttcgccgagactatggaacgatggaacatcgacgagatcaaccgactgcctgactacatgaagcccctgtaccgatgtattctgcgaatgttcgacgactacgagatcgacgccgccaagcagggcaagctgttcgctgtgccctacgctaagcagaccatgaaggaactgtgtcgagcccactgccagggcgtgaagtacactatgggcggacccacctcgtcttttgaggactacatcgtgaacaccatgatcacctctgtgctgtacgtgacctgcgccgccaccattcctggcctgaagtctgcctctaaggaaaccatcgactggttcaagtcgaagcccaagatcatccgagcctctgccatgatctgccgacacctggacgacctgggctctcacgagcgagagtctcagcagggcaccctcctgaccgctctggacatctacatgcgacaccacggtggatctatcaaggaagcccgagagaagttcgaggaactggttgaggacgcctggaaggacctgaacgccgagtggatcaaggacatcaagtctggcgtgtctaaggaagtcgttgaggacttcctgggctacgcccgagccgccgacgtgttctaccgaaactgccgagatggctacgccaaggctcacggcgtgatgaaccccgaggtggacgccctgttcatgtaa |

**Additional file 1: Table S3** The detailed information of genes used in this study.

| Gene | Encoded enzyme | GenBank |
| --- | --- | --- |
| *ERG12* | Mevalonate kinase | YALI0_B16038g |
| *ERG8* | Phosphomevalonate kinase | YALI0_E06193g |
| *ERG19* | Mevalonate diphosphate decarboxylase | YALI0_F05632g |
| *IDI* | IPP isomerase | YALI0_F04015g |
| *GPPS* | GPP synthase | YALI0_D17050g |
| *ERG20* | FPP synthase | YALI0_E05753g |
| *FAA1* | Long chain fatty acyl-CoA synthetase | YALI0_D17864g |
| *PXA1* | Peroxisomal acyl-CoA transporter 1 | YALI0_A06655g |
| *PXA2* | Peroxisomal acyl-CoA transporter 2 | YALI0_D04246g |
| *POX2* | Fatty-acyl-CoA oxidase 2 | YALI0_F10857g |
| *POX3* | Fatty-acyl-CoA oxidase 3 | YALI0_D24750g |
| *MFE1* | Peroxisomal multifunctional enzyme type 1 | YALI0_E15378g |
| *POT1* | 3-ketoacyl-CoA thiolase | YALI0_E18568g |
| *PEX10* | Peroxisomal biogenesis factor 10 | YALI0_C01023g |
| *PAT1* | Acetyl-CoA C-acetyltransferase | YALI0_E11099g |
| *AMPD* | Adenosine monophosphate deaminase | YALI0_E11495g |
| *YHM2* | Mitochondrial citrate carrier | YALI0_B10736g |
| *ACL1* | ATP dependent citrate lyase 1 | YALI0_E34793g |
| *ACL2* | ATP dependent citrate lyase 2 | YALI0_D24431g |
| *TGL3* | Triacylglycerol lipase 3 | YALI0_D17534g |
| *TGL4* | Triacylglycerol lipase 4 | YALI0_F10010g |
| *DGA1* | Diacylglycerol acyltransferase 1 | YALI0_E32769g |
| *DGA2* | Diacylglycerol acyltransferase 2 | YALI0_D07986g |

**Additional file 1: Table S4** The accumulation of by-products at 216 h of fermentation. Data represent the mean ± SD of biological duplicate.

| Substrates | Citric acid  (g/L) | Mannital  (g/L) | Lipids  (mg/g DCW) | Squalene  (mg/g DCW) |
| --- | --- | --- | --- | --- |
| Oleic acid | 21.3 ± 2.8 | 4.9 ± 1.2 | 102.9 ± 26.1 | 1.71 ± 0.6 |
| Waste cooking oil | 28.4 ± 3.6 | 3.7 ± 1.6 | 136.4 ± 22.8 | 5.62 ± 0.5 |
